# CB2-mediated attenuation of nucleus pulposus degeneration via the amelioration of inflammation and oxidative stress in vivo and in vitro

**DOI:** 10.1186/s10020-021-00351-x

**Published:** 2021-08-19

**Authors:** Xiaoqiang Cheng, Jiayi Lin, Zhanghuan Chen, Yubo Mao, Xiexin Wu, Congxin Xu, Jiacheng Du, Zhongchen Dong, Huilin Yang, Feng Zhou, Dechun Geng

**Affiliations:** 1 Department of Orthopaedics, The First Affiliated Hospital of Soochow University, 188, Shi Zi Road, 215006 Suzhou, China; 2 Department of Orthopaedics, Nanjing First Hospital, Nanjing Medical University, 210000 Nanjing, China

**Keywords:** Intervertebral disc degeneration, Nucleus pulposus cell, Inflammation, Oxidative stress, Cannabinoid type 2 receptor

## Abstract

**Background:**

Nucleus pulposus cell (NPC) degeneration is widely accepted as one of the major causes of intervertebral disc (IVD) degeneration (IVDD). The pathogenesis of IVDD is complex and consists of inflammation, oxidative stress, and the loss of extracellular matrix (ECM). Cannabinoid type 2 receptor (CB2) has been shown to be involved in the pathological mechanism of a variety of diseases due to its anti-inflammatory effects and antioxidative stress capacity.

**Method:**

In Vitro, H_2_O_2_ was used to induce degeneration of nucleus pulposus cells, mRNA and protein expression level was determined by RT-PCR and Western Blot, and Immunocytochemical staining were used to detect expression of collagen II, aggrecan, MMP3/13, superoxide dismutase 2 (SOD2) and inducible nitric oxide synthase (iNOS). In vivo, the potential therapeutic effect of CB2 was detected in the rat acupuncture model.

**Result:**

In vitro, we found that the CB2 agonist (JWH133) treatment reduced the oxidative stress level in NPCs induced by hydrogen peroxide (H_2_O_2_) treatment. Furthermore, the expression of inflammatory cytokines was also decreased by JWH133 treatment. We found that collagen II and aggrecan expression was preserved, whereas matrix metalloproteinase levels were reduced. In vivo, we established a rat model by needle puncture. Imaging assessment revealed that the disc height index (DHI) and morphology of IVD were significantly improved, and the disc degeneration process was delayed by treatment of JWH133. Furthermore, immunohistochemical (IHC) staining revealed that JWH133 could inhibit the degradation of collagen II and decrease the expression of MMP3.

**Conclusions:**

The experiment indicates the oxidative stress and inflammatory response of rat NPCs induced by H_2_O_2_ could be inhibited by activating CB2. This study reveals that CB2 activation can effectively delay the development of IVDD, providing an effective therapeutic target for IVDD.

**Supplementary Information:**

The online version contains supplementary material available at 10.1186/s10020-021-00351-x.

## Introduction

Intervertebral disc (IVD) degeneration (IVDD) is a common type of degenerative change in the spine, leading to disc herniation, spinal stenosis, spinal instability, and other degenerative diseases of the spine (Brinjikji et al. 2015; Sun et al. 2017; Dudli et al. 2014; Zhu et al. 2017). It has been reported that IVDD is a major cause of low back pain (LBP), and more than 70 % of the population has suffered from LBP in their lifetime (Walker 2000; Driscoll et al. 2014). Current treatments, either surgical or non-surgical methods, are aimed at relieving the symptoms of IVDD but not its pathogenesis (Mirza and Deyo 2007). However, the pathogenesis of IVDD is complicated and has not yet been elucidated. Therefore, exploring the pathogenesis of IVDD in depth will play a vital role in discovering its potential treatment approaches.

Oxidative stress is the imbalance between the generation of reactive oxygen species (ROS) and the antioxidant defence system (Peluso et al. 2012). In recent years, it has been found that oxidative stress plays a vital role in the occurrence and development of a variety of diseases (Song et al. 2018; Trojnar et al. 2019; Wu et al. 2019; Liu and Shi 2019). The imbalance between the production of ROS and a decreased natural antioxidant defence system in cells leads to inflammatory neutrophil infiltration, increasing the secretion of proteases and the production of ROS. ROS are generated during electron transport chain reactions, delivering electrons, including superoxide anions, hydroxyl radicals, hydrogen peroxide (H_2_O_2_) and nitric oxide, to molecular oxygen in mitochondria. An increasing number of studies have shown that oxidative stress plays a pivotal role in NP degeneration (Dimozi et al. 2015; Tang et al. 2019). Recent studies have shown that the regulation of ROS can delay the degeneration of chondrocytes, which are similar to NPCs (Pan et al. 2017; Kang et al. 2019). In addition, Luo et al. found that the apoptosis rate of human NPCs can be reduced by regulating oxidative stress (Luo et al. 2019). In addition to oxidative stress, the overexpression of inflammatory cytokines, which disrupts the normal physiological function of NPCs, has been detected in degenerative IVD tissue. Some studies have shown that excess inflammation may be one of the pathogenic factors of IVDD, the process of which can be slowed down by inhibiting the inflammatory response in NP tissues (Navone et al. 2017; Ge et al. 2019). Because of the special anatomical environment of the NP, which is wrapped around the annulus fibres and the endplates of the disc, the NP is at increased risk of hypoxia and metabolic disorders. As a result, inflammation and oxidative stress are more likely to occur in NP tissues. The prevention of these risk-associated factors in NPCs may contribute to alleviating the degeneration of NPCs.

Cannabinoid type 2 receptor (CB2), a member of the G protein-coupled receptor family, mainly exists in the cells of the immune system. CB2 mainly participates in regulating cell proliferation, differentiation, apoptosis and cytokine release. Recent studies have shown that CB2 activation inhibits inflammation and oxidative stress (Zhang et al. 2019; Leinwand et al. 2017). Cakir et al. found that CB2 modulates the release of inflammatory cytokines, which further activate the NF-κB signalling pathway, leading to the release of inflammatory cytokines and thus forming a vicious cycle (Cakir et al. 2020). CB2 agonists can inhibit the release and infiltration of inflammatory cytokines. Furthermore, studies have found that CB2 is involved in the regulation of cellular oxidative stress. Mukhopadhyay et al. found that CB2 agonists reduce oxidative stress, downregulate the expression of superoxide-generating enzymes and alleviate the nephrotoxicity induced by cisplatin in rats (Mukhopadhyay et al. 2016). Bai et al. proved that CB2 has a similar protective effect on cartilage in a collagen-induced mouse arthritis model (Bai et al. 2019). NPCs are similar to chondrocytes both in morphology and function. Considering that inflammation and oxidative stress play pivotal roles in NP degeneration, we speculated that CB2 may control and modulate NP degeneration.

In this study, we investigated the effect of CB2 in IVDD with in vivo and in vitro experiments. We found that a CB2 agonist effectively inhibited the oxidative stress and inflammation of NPCs and significantly attenuated the degeneration of NP in the rat needle puncture model. These findings may provide a new therapeutic target for IVDD.

## Materials and methods

### Reagents and antibodies

JWH133, a selective CB2 agonist, was purchased from Tocris Bioscience (Space Import-Export Srl, Milano, Italy). AM630, a selective antagonist, was purchased from Tocris Bioscience. Furthermore, we purchased rabbit anti-rat iNOS (ab15323), SOD2 (ab13533), NOX4 (ab154244), TNF-α (ab6671), IL-1β (ab9722), IL-6 (ab6672), Collagen II (ab34712), Aggrecan (ab3778), MMP3 (ab53015), and MMP13 (ab39012) antibodies from Abcam (Cambridge UK). Secondary antibodies were purchased from Wuhan Amictech Technology Co Ltd. Phosphate-buffered saline (PBS) was purchased from Beyotime (Beyotime Institute of Biotechnology, Jiangsu, China). ROS production was detected by 2′,7′-dichlorodihydrofluorescein diacetate fluorescent probe purchased from Beyotime as well. Fetal bovine serum, Dulbecco’s modified Eagle’s medium/Nutrient Mixture F-12 (DMEM/F12), RIPA protein lysates. Trizol reagent and a BCA protein kit were purchased from Invitrogen (Waltham, MA, USA). Type II collagenase was purchased from Thermo Fisher Scientific. Primary antibodies (Western Blot) were purchased from Abcam and secondary antibodies were purchased from Beijing Zhongshan Jinqiao Biotechnology. Triton-X-100 was purchased from Invitrogen and DAPI was purchased from Beyotime.

### Human samples

Human degenerative NP tissue was obtained from IVDD patients undergoing spinal surgery. Preoperative magnetic resonance imaging (MRI) revealed different degrees of IVDD, which were divided into 3 groups (n = 5) depending on the Pfirrmann grade. Normal NP tissue was obtained from 20-year-old patients undergoing anterior spinal surgery with a burst fracture of the spine (n = 5). Human samples were used for immunohistochemical staining. Written informed consent was obtained from each participant. All experiments with human samples conformed to human ethical guidelines. The study was conducted with the approval of the Ethics Committee of the First Affiliated Hospital of Soochow University (Suzhou China; no: 201801A005).

### Isolation and culture of primary NPCs

Male Sprague-Dawley (SD) rats weighing 250 ± 25 g were sacrificed and sterilized with 75 % ethanol for 15 min. The caudal root (including caudal intervertebral disc tissue) was cut and peeled to fully expose the caudal disc tissue. Subsequently, the cells were moved to a glass panel containing 0.02 % penicillin-streptomycin in PBS for 5 min. The IVD ring was sharpened with a sterile surgical blade and the NP tissue (Co1–Co4) was carefully separated from the annulus fibrosus and placed in PBS containing 1 % penicillin-streptomycin. The tissue was cut into 1 mm^3^ pieces and placed in a separate sterile centrifuge tube. Then, 0.5 % (w/v) type II collagenase solution was added at a volume ratio of 5:1 and the sample were incubated in a water bath at 37 °C for 2 h. Every 20 min, the sample was gently shaken to digest the tissue, which was then centrifuged at 1000 RPM for 6–8 min. The supernatant was removed, and the cells were resuspended in fresh DMEM/F12 complete medium and transferred to a culture flask. The cells were cultured at 37 °C in an incubator with saturated humidity and 5 % CO_2_. Four experimental groups were established. (1) Control group: NPCs were cultured with DMEM/F12 without any other drugs for 12 h. (2) H_2_O_2_ group: NPCs were incubated with DMEM/F12 and H_2_O_2_ (100 µM). (3) JWH133 group: NPCs were cultured with DMEM/F12 supplemented with JWH133 (10 µM) for 3 h and then incubated with DMEM/F12 supplemented with H_2_O_2_ for 12 h. (4) AM630 group: NPCs were cultured with DMEM/F12 mixed with AM630 (10µM) for 3 h and then incubated with DMEM/F12 supplemented with H_2_O_2_ for 12 h.

### Determination of cell viability

To evaluate the effect of different reagents on cell proliferation and differentiation, we plated NPCs (3 × 10^3^/well) in 96-well plates and tested the viability of the NPCs with a Cell Counting Kit-8 (Dojindo Co, Kumamoto, Japan) according to the manufacturer’s instructions. NPCs were treated with H_2_O_2_, JWH133 and AM630 at different concentrations. After incubation at 37 °C for 2 h, the absorbance of the wells was measured at 450 nm by a microplate reader (BioTek Instruments Inc Winooski, VT, USA).

### Real-time PCR

NPCs were dissolved in 1 mL Trizol reagent at room temperature for 5–10 min. Chloroform was added and the cells were shaken for 15 s, incubated on ice for 10 min, and centrifuged at 12,000 RPM for 15 min at 4 °C. The upper liquid fraction was transferred to a fresh Eppendorf tube and shaken vigorously in the same volume of isopropanol (Tclc Chemical, Japan) and incubated on ice for 10 min. After centrifugation at 12,000 RPM for 15 min at 4 °C, the supernatant was aspirated, and 500 µL of 75 % ethanol was added to wash the ribonucleic acid. After another centrifugation step, 10 µL of DEPC (Thermo Scientific, CA, USA) water was added to resuspend the RNA and the sample was incubated for 8 min at 58 °C in a water bath; subsequently, the RNA concentration was measured using a NanoDrop 2000 (Thermo Fisher Scientific, Waltham, MA, USA). For PCR amplification, a 20 µL reaction mixture consisting of 10 µL SYBR Green Real-Time PCR Master Mix (Thermo Fisher Scientific, Waltham, MA, USA), 1 µL of each primer (10 µmol/L), 1 µL of cDNA, and 8 µL of RNase-free distilled water (Invitrogen). The PCR conditions were as follows: (1). initial denaturation for 10 min at 95 °C. (2). 15 s at 95 °C for 40 cycles. (3). 1 min at 60 °C. Cycle threshold (CT) values were standardized to those of glyceraldehyde 3-phosphate dehydrogenase (GAPDH). The data are presented as the fold change (2^−ΔΔCT^). The experiment was repeated three times. Primers details are shown in Table 1.

**Table 1:** Patient information

Pfirrmann grade	Patient	Age	Sex	Segment
I	#1	21	M	T10–11
	#2	24	M	T8–T9
	#3	24	M	T8–T9
	#4	22	M	T10–T11
	#5	25	M	T12–L1
III	#1	39	F	L4–5
	#2	44	M	L5–S1
	#3	38	M	L4–5
	#4	42	F	L5–S1
	#5	42	F	L4–5
IV	#1	44	F	L5–S1
	#2	56	M	L5–S1
	#3	67	M	L4–5
	#4	59	M	L5–S1
	#5	61	M	L5–S1
V	#1	77	F	L4–5
	#2	68	M	L5–S1
	#3	72	M	L5–S1
	#4	69	F	L4–5
	#5	69	F	L4–5

### Western blot assay

Cells were harvested and lysed on ice using 10 µL of radioimmunoprecipitation assay (RIPA) buffer supplemented with phenylmethanesulfonyl fluoride (PMSF). An equivalent amount of protein was separated by sodium dodecyl sulfate-polyacrylamide gel electrophoresis (SDS-PAGE) and transferred to a polyvinylidene fluoride (PVDF) (PVDF) membrane (Bio-Rad). After transfer, the PVDF membrane was rinsed with deionized water, buffer containing 5 % skim milk (Yili, Inner Mongolia, China) was added, and the membrane was incubated at room temperature for 1 h. Then, the membrane was incubated with a primary antibody (1:1000) overnight at 4 °C. After the membrane was washed with TBST and incubate with secondary antibody (1:5000) for 1 h in room temperature. After washing the membrane with TBST for 3 times, the protein signals were detected using a chemiluminescence kit and a gel imaging device (Thermo Scientific, CA, USA). The dilution ratio of primary antibodies was listed as follow: iNOS (1:1000), SOD2 (1:5000), NOX4 (1:1000), TNF-α (1:1000), IL-1β (1:5000), IL-6 (1:1000), Collagen II (1:1000), Aggrecan (1:1000), MMP3 (1:100), and MMP13 (1:3000).

### Immunocytochemical staining

NPCs were cultured in 24-well plates. After being washed with PBS, the cells were fixed with 4 % paraformaldehyde and blocked with Triton-X-100 for 10 min and then incubated with 10 % normal goat serum/1 % bovine serum albumin/0.3 M glycine in 0.1 % PBS-Tween for 2 h to block non-protein-protein interactions. The cells were incubated overnight at 4 °C with iNOS (1:200), SOD2 (1:200), Collagen II (1:200), Aggrecan (1:200), MMP3 (1:200) and MMP13 (1:200) antibodies. Then, the cells were incubated with secondary antibodies for 1 h. DAPI was used to counterstain cell nuclei (blue) for 30 min at a concentration of 1.43 µM.

### Measurement of ROS production

NPCs (1 × 10^5^ cells/well) were plated in 24-well plates, which were pretreated with JWH133 or AM630 and incubated with 75 µM H_2_O_2_. Then, the NPCs were incubated with 10 µM 2’,7’-dichlorodihydrofluorescein diacetate for 30 min away from light. Flow cytometry and fluorescence microscopy (Olympus Inc, Tokyo, Japan) were used to evaluate ROS-mediated fluorescence. Positive cells were marked green.

### Establishment of the rat needle puncture model

A total of 40 (n = 40) SD rats (male, weighing 400 ± 20 g) were provided by the Animal Experimental Centre of Soochow University. MRI scans and X-ray examination confirmed that there was no congenital deformity or disc degeneration in the caudal vertebra. The rats were randomly divided into 4 groups by the digital table method: normal group, vehicle group and 2 experimental groups, with 10 rats in each group. The Co 7/8 IVD was located by operative X-ray. The rats were weighed and anaesthetized intraperitoneally with 10 % chloral hydrate (3.5 mL/kg). The rats were fixed on the operating table, and the Co 7/8 IVD was punctured with a 21 G injection needle. The tip of the needle was inserted perpendicular to the rat tail and retracted 30 s later. After the operation, the rats were free to move around in cages without dietary restrictions, and urinary retention and infection were closely observed.

The vehicle group was not treated with JWH133 or AM630. The experimental group was divided into 2 groups (JWH133 and AM630). Before IVD injection, the rat tails were dissected at Co 7/8, and the distance between the skin and the intervertebral nucleus was measured (approximately 7 mm). After IVD models were successfully established, JWH133 (30 µg/ml) and AM630 (30 µg/ml) were respectively subcutaneous injected weekly with 2µL drugs. Vehicle group was punctured without any further treatment. Ctrl (Sham) group was established with incision and suture of skin (Co7-8 level) with surgical knife. No rats died during modeling or before sampling and the dropout rate is 0.

### X-ray and MRI examination

At 1, 2, 3 and 4 weeks after the operation, 2 rats were randomly selected from each group to undergo radiological examination. The rats were anaesthetized intraperitoneally with 10 % chloral hydrate (3.5 mL/kg), and their tails were placed in a straight line on a molybdenum radiography device (GE Healthcare, Chicago, IL, USA). The X-ray was taken at a distance of 66 cm from the collimator to the film. Then, the rats underwent a 1.5 T magnetic resonance scan (Philips Eclipse) using T2-weighted sagittal parameters: TR/TE: 3500/102 ms, FOV: 15.0, thickness: 3 mm, interval: 0 mm. The degree of disc degeneration was assessed by the signal intensity of the T2-weighted image of the disc.

### Histological examination

After the imaging examination was completed, the rats were sacrificed, the Co7-8 IVD was extracted completely, and 4 % paraformaldehyde was used to fix the tissue for 48 h. The disc was decalcified with 10 % EDTA(Invitrogen) for 4 weeks and then cut into 5 horizontal sections of 5 μm thickness. The sections were used to conduct haematoxylin-eosin (H&E) and Safranin O Fast Green staining. The disc morphology was observed and scored by light microscopy. The histological grade was determined according to the method described by Masuda (2005).

### Immunohistochemical staining

The expression of collagen II and MMP3 was detected by immunohistochemistry. The sections were dewaxed in xylene, dehydrated in a graded series of ethanol solutions, and incubated with 3 % H_2_O_2_ at 37 °C for 10 min. The sections were then washed with PBS for 5 min, boiled in 0.01 M citrate buffer, antigen extracted (95 °C, 15–20 min), and blocked with goat serum at 37 °C for 10 min. The sections were subsequently incubated with primary antibodies (anti-collagen II and anti-MMP3, Abcam, USA) overnight at 4 °C, followed by biotin-labelled secondary antibodies (Bioworld) for 30 min at 37 °C. The sections were re-stained with haematoxylin and observed by light microscopy. The results were analysed by Image-Pro Plus 6.0 (Media, Cybernetics Bethesda, MD, USA). The dilutions of antibodies are as follows: CB2 (1:50), Collagen II (1:100), MMP3 (1:100).

The quantification of immunohistochemical staining was accessed with Image-pro plus (IPP) software. Selecting area of interest and measuring the OD value. then, choose and measure the area of the valid statistical area. Calculating the average optical density of the selected region. Finally, calculating the mean value and standard deviation of optical density of each experiment group. Statistical analysis was used to access statistical difference.

### Ethics statement

The experimental protocol in this study was conducted with the approval of the Ethics Committee of the First Affiliated Hospital of Soochow University (Suzhou China; no: 201801A005). All the experimental animals were raised and operated on in accordance with the Helsinki Declaration (1964) and the Laboratory Animal Guidelines for the Ethical Review of Animal Welfare (GB/T 35,892 − 2018, China).

### Statistical analysis

We used SPSS 17.0 for statistical analysis. One-way ANOVA was used for accessing differences among groups. Comparison between two groups was accessed by least square difference (LSD) method. The mean difference is expressed as a 95 % confidence interval. Values of p < 0.05 were considered statistically significant.

## Results

### CB2 expression is reduced in degenerated NP tissue

**Fig. 1:**
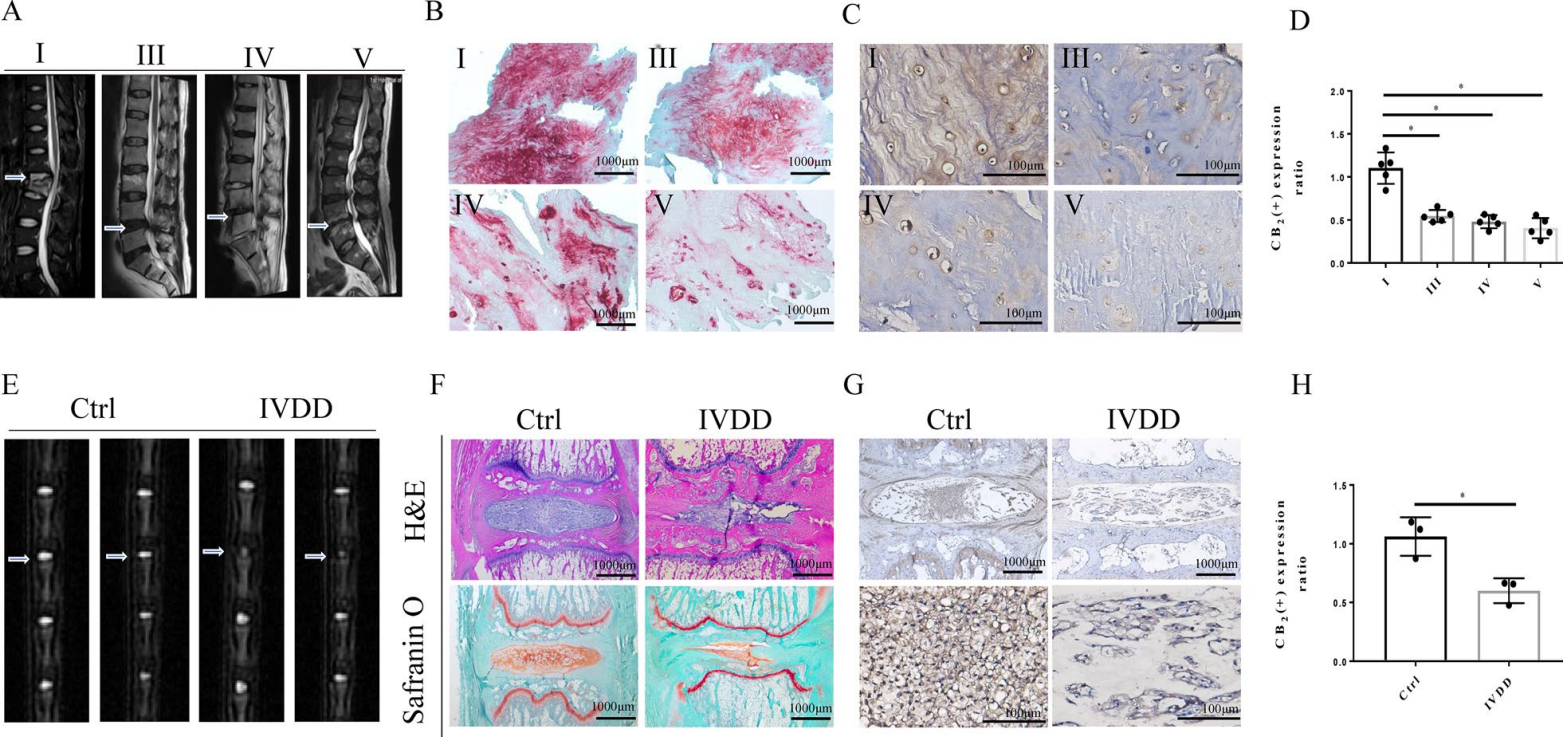
Low expression of CB2 was observed in both human and rat degenerative NP tissue. **A** Preoperative MRI of participants, who were divided into 4 groups (level: I, III, IV, V) according to the Pfirrmann classification. **B** Safranin O staining of participant NP tissues; scale bar, 1000 μm. **C** Representative specimens of immunohistochemical CB2 staining in the 4 separate groups (level: I, III, IV, and V) of participant NP tissues; scale bar, 100 μm. **D** Quantitative analysis of IHC staining of CB2 (*p < 0.05). **E** Representative MRI showing the signal intensity of IVD in rats. **F** H&E and safranin O staining, scale bar, 1000 μm. **G** Representative specimens of paraffin sections with IHC of CB2 in normal and IVDD rats. **H** Quantitative analysis of CB2 IHC staining (*p < 0.05). (n = 5 Independent experiments)

As shown in Fig. 1A, we collected normal and degenerated NP tissue from patients and found different levels of IVDD based on the Pfirrmann grade. Safranin O staining revealed a mass of normal NPCs (chondrocyte-like cells) in the Pfirrmann grade I group. However, the NPCs showed different degrees of decline consistent with the level of degeneration. NP tissue was subsequently substituted by hypocellular fibrocartilaginous tissue (Fig. 1B). To explore the relationship between CB2 and IVDD, we first evaluated the expression of CB2 in human NP tissues. IHC staining suggested that the number of CB2-positive cells was negatively correlated with the level of IVDD. Lower levels of CB2 were observed in human degenerated NP tissues than normal NP tissues (p < 0.05; Fig. 1C, D). In addition, we established a rat by needle puncture-induced IVDD model and more severe degeneration was observed in the degenerative group compared with the normal group (Fig. 1E). H&E and safranin O staining showed that the height of the IVD was decreased and the NP morphology was disordered (Fig. 1F). Furthermore, decreased CB2 expression was found in the degenerative group, which was similar to that in humans (p < 0.05; Fig. 1G, H). All these results indicate that CB2 may be closely associated with IVDD.

### CB2 participates in regulating the H_2_O_2_-induced degeneration of NPCs

**Fig. 2:**
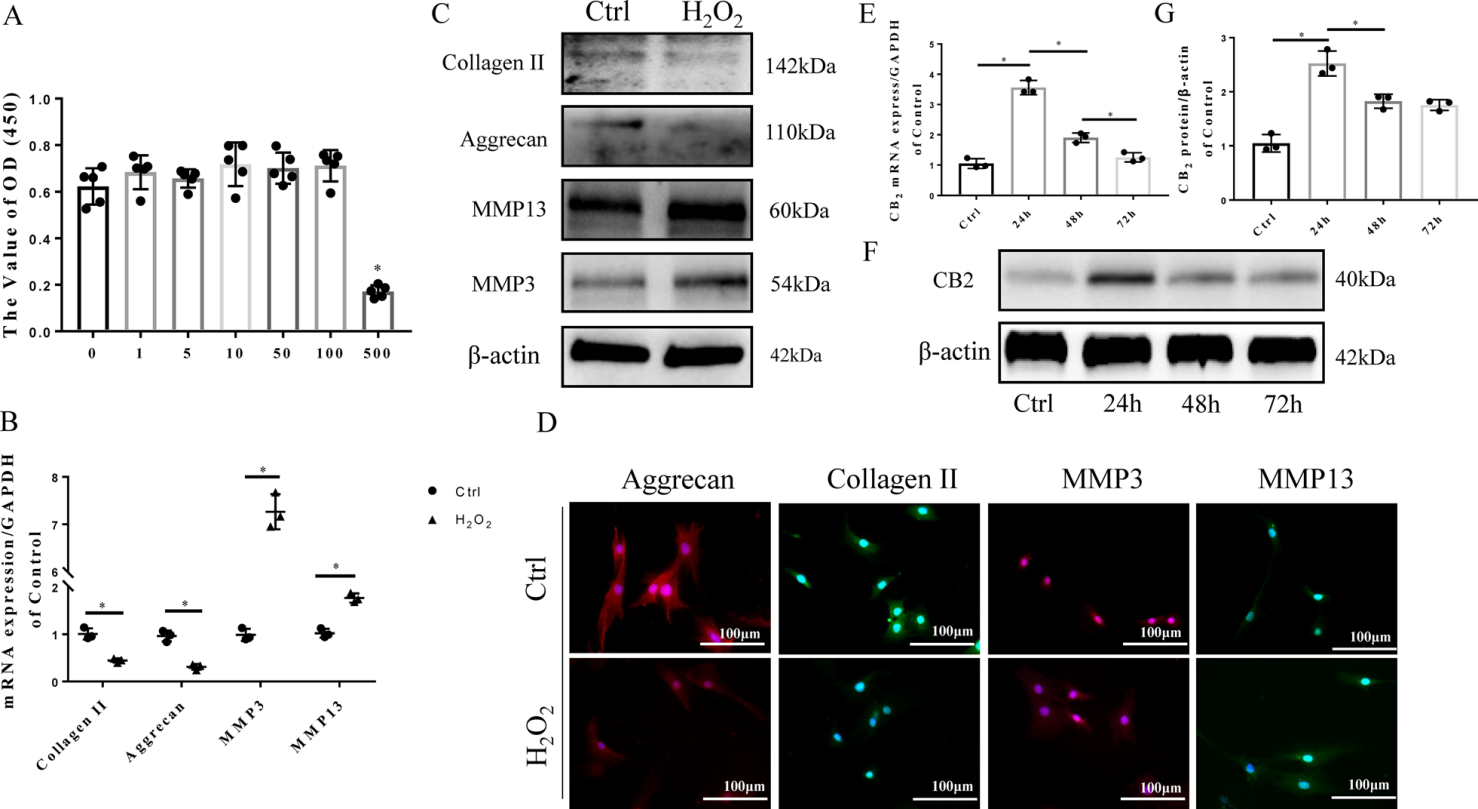
H_2_O_2_ induces NPC degeneration and affects CB2 expression in NPCs. **A** CCK-8 results showing that cell viability is not affected by H_2_O_2_ at a concentration of 100 µM (*p < 0.05 compared with H_2_O_2_ 100 µM, 12 h). **B** RT-qPCR results for aggrecan, collagen II, MMP3 and MMP13 (*p < 0.05). **C** Western blot results of collagen II, aggrecan, MMP3 and MMP13. **D** Representative ICC image of collagen II, aggrecan, MMP3 and MMP13; scale bar, 100 μm. **E** RT-qPCR results of CB2 at different time points after treatment (*p < 0.05). **F** Western blot results of CB2 at different time points after H_2_O_2_ treatment. **G** Quantitative analysis of the Western blot (* p < 0.05) (n = 3 Independent experiments)

To explore the molecular mechanisms underlying the effect of CB2 on NP degeneration, H_2_O_2_ was used to induce NPC degeneration. As shown in Fig. 2A, H_2_O_2_ had no cytotoxicity at a concentration of 100 µM (p < 0.05). The mRNA levels of the NP matrix components aggrecan and collagen II were reduced by H_2_O_2_ treatment. Furthermore, the mRNA levels of MMP3 and MMP13, degeneration markers, were significantly increased (p < 0.05; Fig. 2B). The Western blot results showed that a small amount of MMP3 and MMP13 protein was present in normal NPCs and increased significantly after H_2_O_2_ treatment. Furthermore, our results proved that collagen II and aggrecan expression markedly decreased following H_2_O_2_ treatment (p < 0.05; Fig. 2C), suggesting that H_2_O_2_ induces the degeneration of NPCs. ICC results also confirmed the degeneration of NPCs (Fig. 2D). Interestingly, we found that the protein expression of CB2 was significantly increased by ~ 2.8-fold and that mRNA expression was increased by ~ 3.9-fold within the first 24 h under H_2_O_2_ intervention conditions (p < 0.05). Subsequently, the CB2 expression decreased over the next 48 h, indicating that CB2 exerts a protective effect during the early stage of NPC degeneration (Fig. 2E–G).

**Fig. 3:**
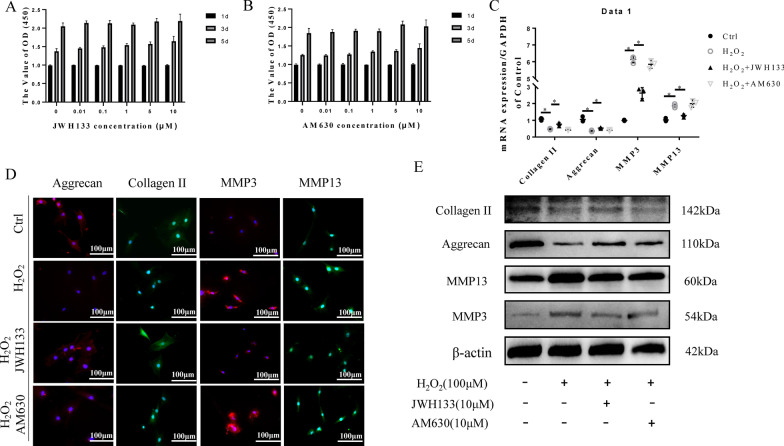
CB2 activation attenuates the degeneration of NPCs. **A**, **B** CCK-8 results showing that both JWH133 and AM630 have no toxicity in NPCs at concentrations under 10 µM **C** RT-qPCR results for collagen II, aggrecan, MMP3 and MMP13 (*p < 0.05). **D** Representative image of ICC staining for collagen II, aggrecan, MMP3 and MMP13 in NPCs; scale bar, 100 μm. **E** Western blot results for collagen II, aggrecan, MMP3 and MMP13. (n = 3 Independent experiments)

To define the function of CB2 in NP degeneration, we pre-treated NPCs with a CB2 agonist (JWH133) or inhibitor (AM630) and then stimulated them with H_2_O_2_. As shown in Fig. 3A, B, the CCK-8 results showed that the viability of the NPCs was not affected by treatment with either JWH133 or AM630 at a concentration of 10 µM (p > 0.05), which was used in subsequent experiments. The decreased expression of NPC functional factors and increased expression of matrix-degrading enzymes was detected in the vehicle group (p < 0.05). However, treatment with JWH133 reversed the changes in these functional and degradative factors (p < 0.05; Fig. 2C, E). Furthermore, the quantitative analysis of Western blot showed statistical differences (p < 0.05, Additional file 1:  Fig. S1A–D). Interestingly, compared with the vehicle group of NPCs, the AM630-treated group showed no significant changes in the above factors (p > 0.05). Immunocytochemical (ICC) staining also suggested that JWH133 inhibits the degeneration of NPCs (p < 0.05; Fig. 3D). Meanwhile, we also demonstrated that AM630 did not cause phenotypic change of NPCs (Additional file 2: Fig. S2). These results prove that CB2 activation can attenuate the degeneration of NPCs induced by H_2_O_2_.

### CB2 activation attenuates H_2_O_2_−induced oxidative stress and inflammation in vitro

**Fig. 4:**
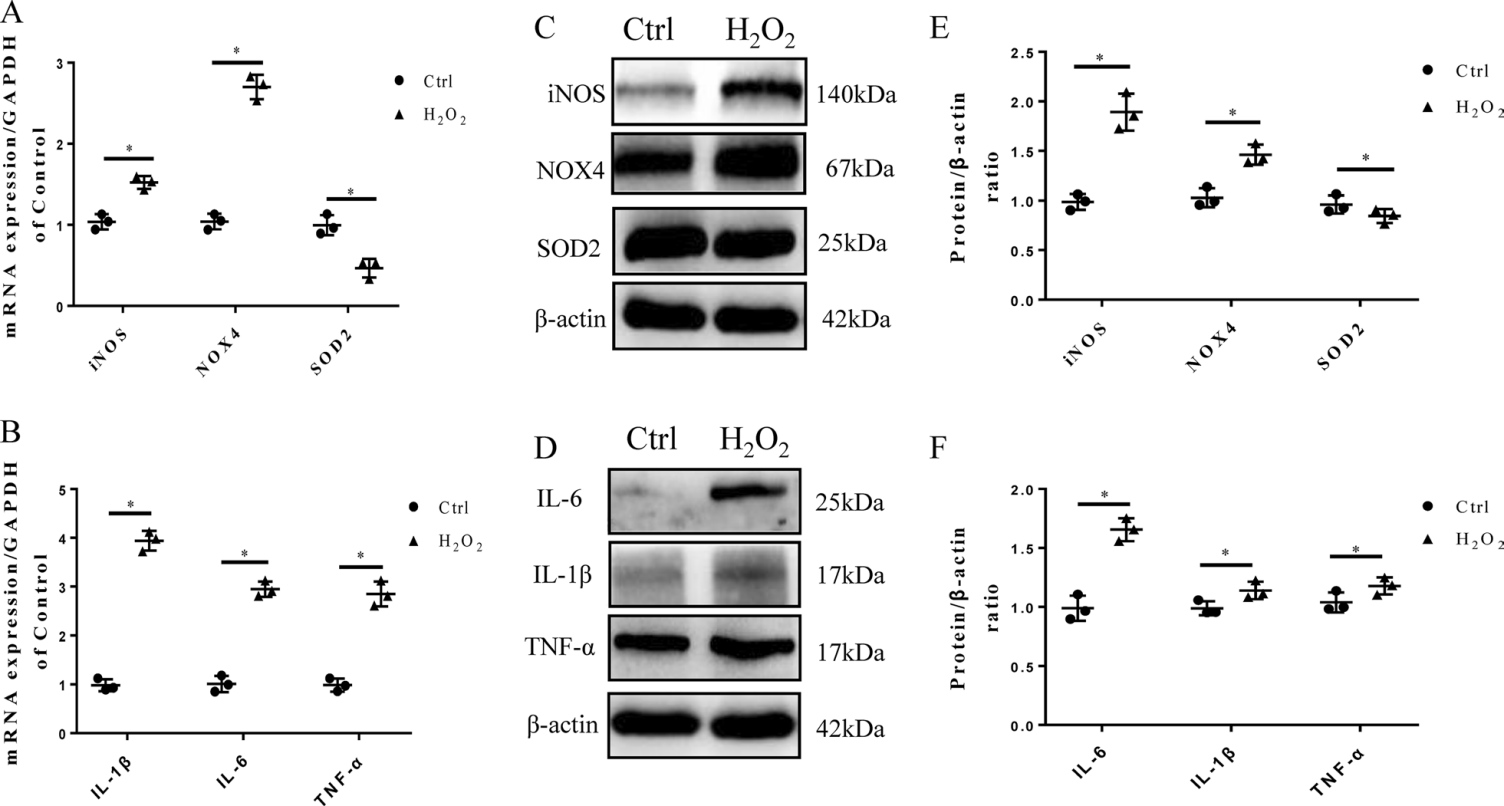
H_2_O_2_ induces high levels of oxidative stress in NPCs. **A** RT-qPCR results of the oxidative stress factors iNOS, SOD2 and NOX4 (*p < 0.05). **B** RT-qPCR results of the inflammatory cytokines IL-1β, IL-6, and TNF-α (*p < 0.05). **C** Western blot results for iNOS, SOD2, and NOX4. **D** Western blot results for IL-1β, IL-6, and TNF-α. **E** Quantitative analysis of Western blot for iNOS, SOD2, NOX4 (*p < 0.05). **F** Quantitative analysis of Western blot for IL-1β, IL-6, TNF- α (*p < 0.05). (n = 3 Independent experiments)

We next clarified whether H_2_O_2_ causes oxidative stress and inflammatory damage to NPCs. Our RT-qPCR and Western blot results demonstrated that the expression of iNOS and NOX4 in NPCs increased in response to H_2_O_2_ treatment, whereas the level of SOD2 decreased (p < 0.05, Fig. 4A). Furthermore, H_2_O_2_ increased the levels of the inflammatory cytokines IL-1β, IL-6, and TNF-α (p < 0.05; Fig. 4B). All these results were also detected by Western blot (Fig. 3C–D). The quantitative analysis of western blot revealed statistical differences (p < 0.05, Fig. 4E, F).

**Fig. 5:**
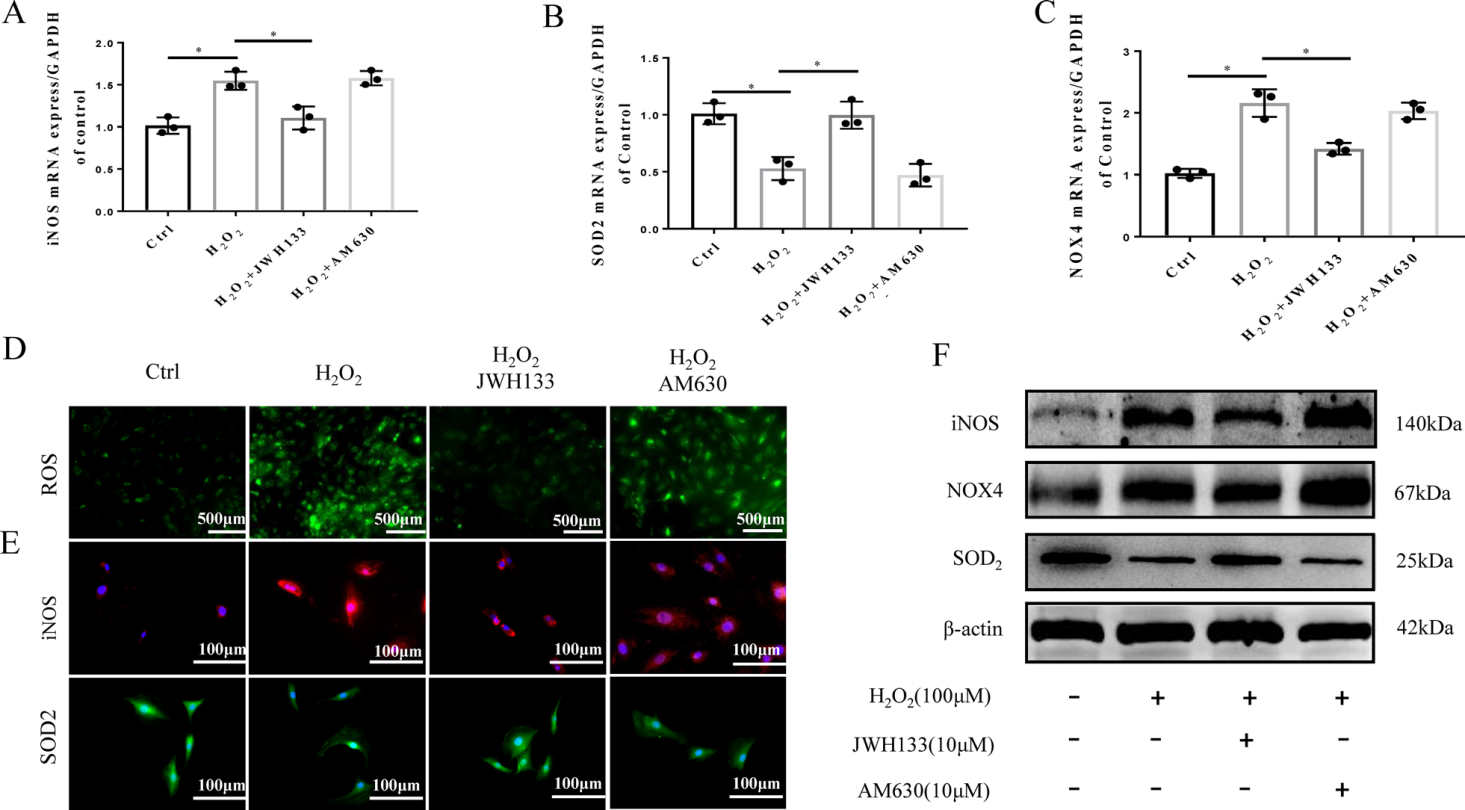
CB2 activation ameliorates oxidative stress in NPCs. **A**–**C** RT-qPCR results for iNOS, SOD2 and NOX4 (*p < 0.05). **D** Fluorescence images showing the levels of ROS in the different groups; scale bar, 200 μm. **E** Representative image of ICC staining for iNOS and SOD2; scale bar, 100 μm. **F** Western blot results for iNOS, SOD2, and NOX4. (n = 3 Independent experiments)

As expected, compared with the H_2_O_2_ group, the JWH133-treated group had significantly inhibited ROS production (Fig. 5D). The quantitative analysis of ROS showed statistical significance (Additional file 1: Fig. S1H). Our results showed that treatment with JWH133 reversed the changes in oxidative stress factors, including iNOS, SOD2 and NOX4, which were increased by H_2_O_2_ treatment (p < 0.05; Fig. 5 A–C, F). The quantitative analysis showed statistical significance (p < 0.05 Additional file 1: Fig. S1E–G). However, the experimental data of these oxidative stress factors in the AM630 and H_2_O_2_ groups were not significantly different (p > 0.05). To further confirm the antioxidative stress effect of CB2, we measured the expression of SOD2 and iNOS by ICC (Fig. 5E). The increased oxidative stress induced by H_2_O_2_ was reversed by JWH133 treatment (p < 0.05). However, there were no similar or opposite results in the JWH133 group compared with the AM630 group, indicating that the inhibition of CB2 may not be involved in the regulation of oxidative stress. These results suggest that CB2 activation negatively regulates oxidative stress and thus exerts a protective effect on NPC degeneration.

**Fig. 6:**
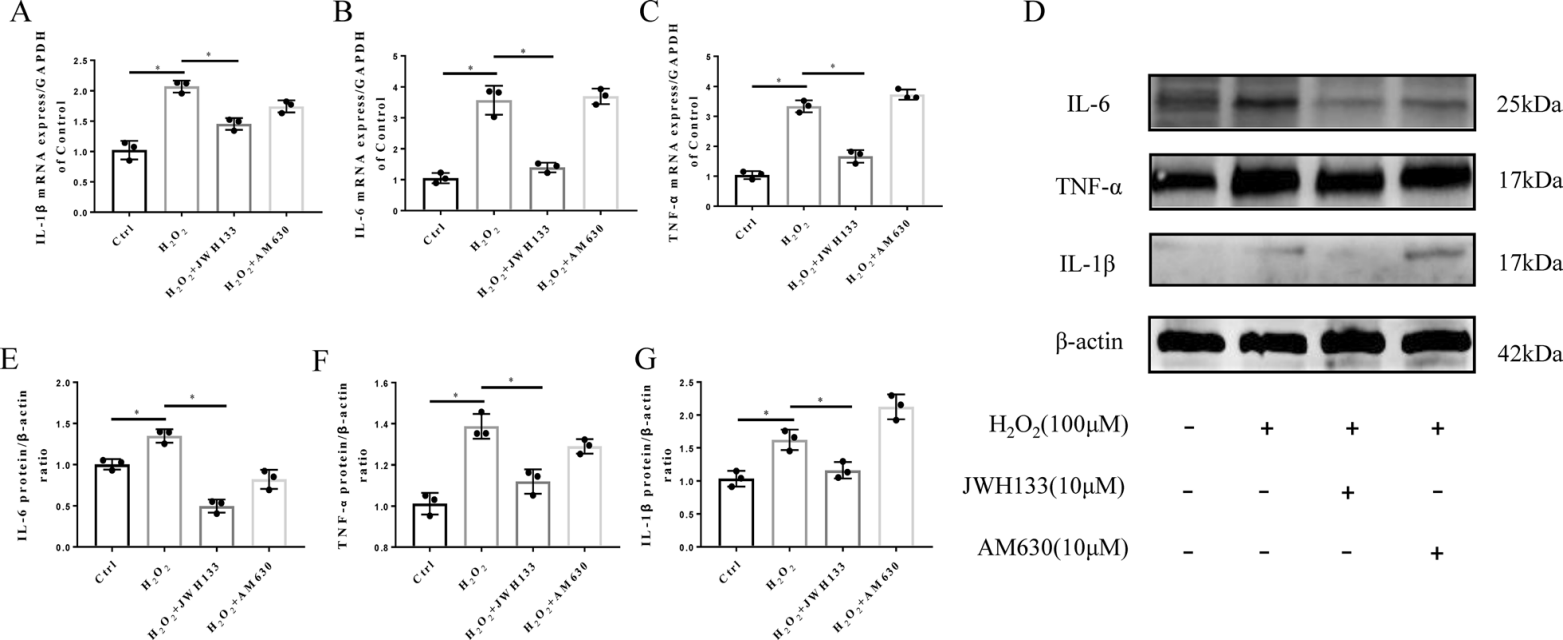
CB2 activation ameliorates inflammation in NPCs. **A**–**C** RT-qPCR results for the inflammatory cytokines IL-1β, IL-6, and TNF-α (*p < 0.05). **D** Western blot results for IL-1β, IL-6, and TNF-α. **E**–**G** Quantitative analysis of the Western blot (p < 0.05). (n = 3 Independent experiments)

We have already demonstrated that CB2 can ameliorate NPC degeneration by reducing the level of oxidative stress. Oxidative stress is closely associated with inflammation, and oxidative stress is usually accompanied by an inflammatory response. To clarify whether the anti-inflammatory effect of CB2 is involved in the mechanism by which it mitigates IVDD, RT-qPCR and Western blot analysis were used. The results indicated that H_2_O_2_ increased the release of relevant inflammatory cytokines (IL-1β, IL-6 and TNF-α), which were significantly decreased after JWH133 treatment (p < 0.05; Fig. 6 A–D). Furthermore, the quantitative analysis of Western blot revealed statistical significance (p < 0.05, Fig. 6E–G). All these results prove the anti-inflammatory effect of CB2 in NPCs.

### CB2 activation attenuates IVDD in vivo

**Fig. 7:**
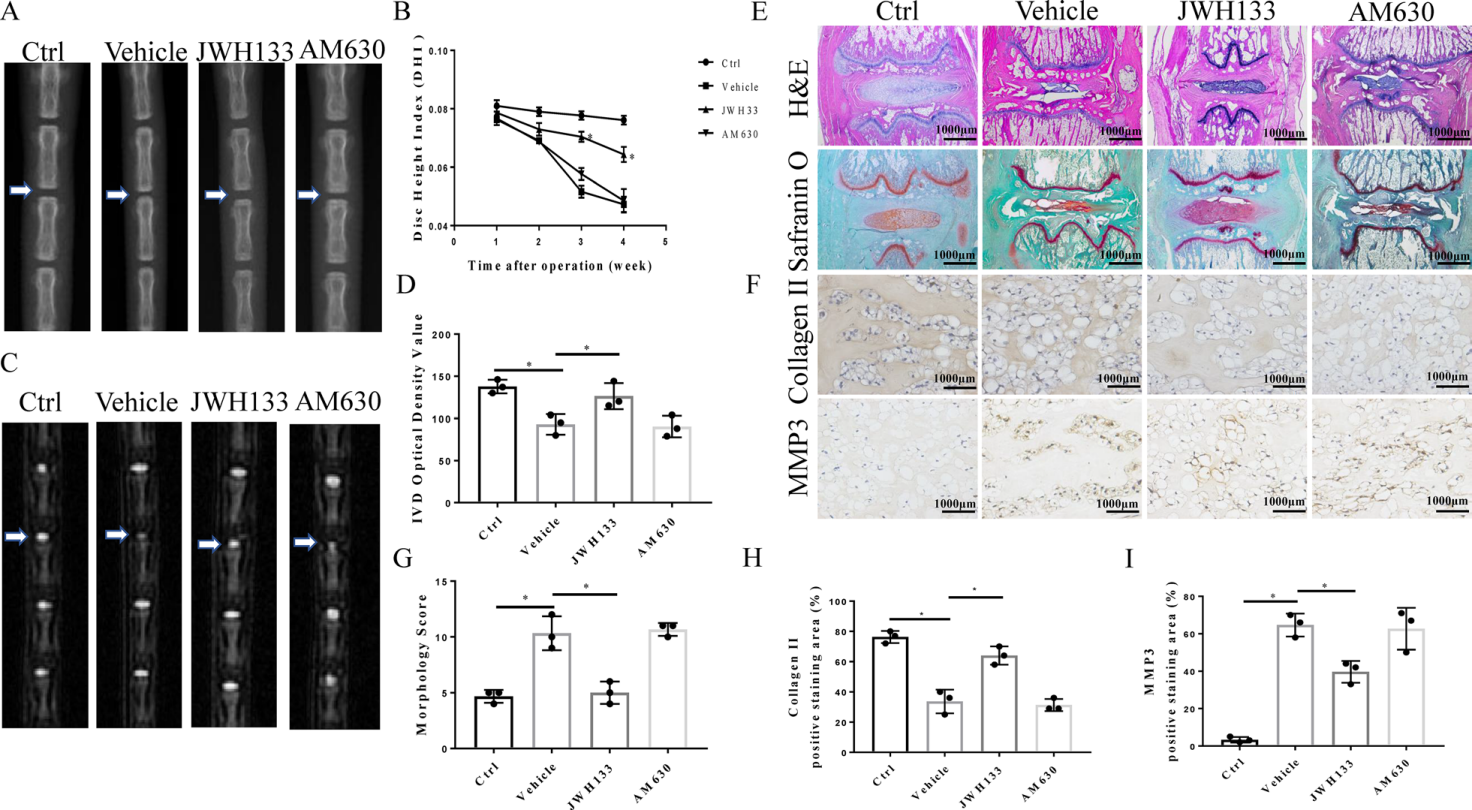
CB2 Activation alleviates IVDD in vivo. SD rat coccygeal Co 7–8 vertebrae were punctured and treated with JWH133 and AM630. **A** Representative X-ray images of rats in the 4 groups (Control, Vehicle, JWH133, and AM630). **B** Quantitative analysis of DHI at different time points after the operation (*p < 0.05 compared with Vehicle group). **C** Representative MRI image of rats in the 4 groups. **D** Quantitative analysis of IVD optical density (*p < 0.05). **E** H&E and safranin O staining of the 4 experimental groups, scale bar, 1000 μm. **F** Representative IHC images of collagen II and MMP3 in the 4 experimental groups, scale bar, 1000 μm. **G** Quantitative statistics of the histological scores. **H**, **I** Quantitative analysis of the positive staining area for collagen II and MMP3 (p < 0.05). (n = 10 Independent experiments)

As shown in Fig. 7A, the height of the IVD in the vehicle group decreased significantly after needle puncture, and this decline was attenuated by JWH133 treatment. The quantitative analysis showed that the disc height index (DHI) decreased from 0.082 to 0.049 in the vehicle group. However, the DHI increased by ~ 1.4-fold in the JWH133 group. Furthermore, there was no significance between the AM630 and vehicle groups (p > 0.05). In the first two weeks after operation, we found no significant improvement in DHI after JWH133 treatment (p > 0.05), until the third week, when statistical analysis showed a significant difference between the two groups (p < 0.05), and the effect of JWH133 treatment was more apparent at the fourth week (p < 0.05 Fig. 7B). MRI scans revealed that the disc signal intensity of the JWH133 group was greater than that of the vehicle group (Fig. 7C). The quantitative analysis of IVD optical density confirmed the therapeutic effect of JWH133 as well (p < 0.05, Fig. 7D). These imaging examinations demonstrated that IVDD was mitigated by JWH133 treatment. However, there was no significant difference in the disc height and signal in the AM630 group, which was consistent with that in the vehicle group (p > 0.05).

H&E and Safranin O Fast Green staining (Fig. 7E) showed that IVDD was not evident in the JWH133 group, and morphologically the NP was more intact and regular than that in the vehicle group. The quantitative analysis indicated that the histological score increased by ~ 1.9-fold in the vehicle group and decreased from 9.2 to 5.5 after treatment with JWH133 (Fig. 7G). However, no significant difference was found between the vehicle group and the AM630 group. Furthermore, IHC staining of the NP tissue revealed that the expression of collagen II was obviously increased, whereas the level of MMP3 was decreased in the JWH133 group compared with the vehicle group (p < 0.05, Fig. 7F). We observed a dramatic improvement in the JWH133 group compared with the vehicle group in the quantitative analysis. Collagen II expression increased by ~ 1.7-fold, whereas MMP3 levels decreased from 80 to 32 % (Fig. 7H, I), indicating that the loss of ECM was partially inhibited and thus delayed IVDD.

## Discussion

IVDD is one of the most common problems that affects the health of the population worldwide and causes a large socioeconomic burden. To further improve the therapeutic effect of IVDD, promote drug development and improve therapeutic strategies, it is necessary to understand the pathological mechanism of IVDD. Current studies suggest that inflammation and oxidative stress contribute to NPC degeneration (Wang et al. 2018; Hwang et al. 2018). In other words, the inhibition of inflammation and oxidative stress in NPCs can be an effective treatment approach. We found that CB2 is involved in regulating the degeneration of the NP, and the degeneration of NPCs can be significantly ameliorated by activating CB2.

CB2 is one of the target receptors of the endocannabinoid system and binds with anandamide and 2-arachidonoylglycerol to exert physiological effects. CB2 is mainly expressed in neutrophils, macrophages, T and B cell subsets and epithelial cells and is thus mainly associated with immune function (Galiegue et al. 1995). Although we found a significant decrease in CB2 expression in vivo, interestingly, we observed some differences in vitro. We induced the degeneration of NPCs with H_2_O_2_ and detected CB2 mRNA and protein levels. We found that the level of CB2 increased in the first 24 h of H_2_O_2_ treatment, but the mRNA and protein levels decreased as the treatment time increased (24–74 h). This phenomenon deserves our consideration. Trojnar et al. found that in a bile duct ligation-induced rat model of hepatorenal syndrome, the expression of CB2 increased as the disease progressed (Trojnar et al. 2019). which is different from our study results. We believe that in the early stage of the disease, the NPCs are still active. To resist the adverse effects of harmful stimuli, NPCs spontaneously increase the level of CB2, increase the binding rate of endocannabinoids to their receptors, and play anti-inflammatory and antioxidative stress roles. As the H_2_O_2_ treatment continues, the protective function of the NPCs becomes exhausted in the subsequent stages; the previously overexpressed CB2 becomes inactivated or the sensitivity becomes reduced, leading to a significant decrease in the CB2 level.

It is widely accepted that oxidative stress plays a vital role in NP degeneration (Zhou et al. 2019; Chen et al. 2014). The imbalance between oxidative stimulation and antioxidant capacity leads to the generation of oxidative stress. Giacoppo et al. found that cannabinol inhibits the oxidative stress induced by H_2_O_2_, and in vitro experiments have proved that cannabigerol enhances the antioxidant stress ability of cells by activating CB2 and upregulating the expression of SOD-1 (Giacoppo et al. 2017). We found that the activation of CB2 can also upregulate the expression of SOD-2. We speculate that SOD-1 and SOD-2 jointly contribute to the improvement of the antioxidant capacity. In addition, Mukhpadhyay et al. hypothesised that cisplatin chemotherapy causes renal toxicity mostly through excessive oxidative stress reactions and found that the activation of CB2 obviously reduced the expression of NOX4 and iNOS and relieved oxidative stress damage (Mukhopadhyay et al. 2010). Similarly, we found that CB2 activation can also reduce the expression of NOX4 and iNOS in NPCs, improving intracellular mitochondrial function. Furthermore, activation of CB2 was proved to downregulate overproduction of ROS, which ameliorate perturbation of cell function and homeostasis, like inhibiting synthesis of ECM or increasing the expression of matrix degrading enzymes (Krupkova et al. 2016).

Inflammation often occurs with oxidative stress and we found that H_2_O_2_ can also induce an inflammatory response in NPCs (Dandekar et al. 2015), which is consistent with the research results of Saha et al. (2019). Rajesh et al. found that TNF-α can induce the activation of NF-κB and RhoA in human coronary endothelial cells and upregulate the expression of the adhesion molecules ICAM-1 and VCAM-1, which enhance the adhesion of monocytes to endothelial cells (Rajesh et al. 2007). The above effects can be attenuated by activating CB2. Furthermore, Fechtner et al. found that CB2 activation significantly inhibits IL-1-induced IL-6 and IL-8 production in human rheumatoid arthritis synovial fibroblasts (Fechtner et al. 2019) and our study results suggest that the production of TNF-α, IL-1β, and IL-6 is significantly inhibited by CB2 activation in NPCs. Increased inflammatory responses can accelerate the ageing and apoptosis of NPCs and accelerate the process of degeneration. CB2 protects the physiological function of NPCs due to its anti-inflammatory effects. Although the therapeutic effect of CB2 cannot completely rescue degenerated NPCs in rats, we cannot deny the role the CB2-induced protective effect plays in delaying the degeneration of NPCs via its anti-inflammatory activity.

However, there are still some limitations in the present study. One of the shortcomings of the experiment is that some influence factors (sex, age, diseases) were not considered in the collection of human samples, which may lead to differences in the levels of oxidative stress and inflammation of nucleus pulpous in individual intervertebral disc specimens. This is also a problem that our research group will focus on in future studies. Second, cell death is a vital part in IVDD, whether CB2 can regulate apoptosis of NPC is still unclear. IVDs consist of the annulus fibrosus, NP and cartilage endplate. It is unclear whether CB2 has a similar effect in the annulus fibrosus and endplate tissues. We are currently addressing this knowledge gap and will continue to do so in the future. Furthermore, there are still some differences between acute injury (needle puncture) and the natural degeneration of the NP. However, the needle puncture model is still the most widely used in the world due to its high economic benefits, convenience and low infection risk (Qian et al. 2019).

## Conclusions

The current study shows that CB2 activation can enhance the antioxidant and anti-inflammatory capacity of NPCs to prevent or ameliorate disc degeneration in vivo and in vitro. These findings suggest that CB2 may be an efficacious therapeutic target for IVDD that acts via suppressing inflammation and oxidative stress.

## Supplementary Information

**Additional file 1: Figure S1.** Quantification of western blot. A-D: quantification of degenerative markers in western blot (Fig. 3, E), E-G: quantification of oxidative stress markers (Fig. 5, F), H: CB2 mRNA expression level in different time points (0, 24, 48, 72, 96, 120 h). (*p < 0.05).

**Additional file 2: Figure S2.** Inhibition of CB2 do not cause phenotypic change of NPCs. A: western blot results for collagen II, aggrecan, MMP3, MMP13. B: quantification of western blot. C-F: mRNA expression level of collagen II, aggrecan, MMP3 and MMP13. (NS: p > 0.05).

## Data Availability

The datasets during current study are available from the corresponding author on reasonable request.
