# Supplementary figures and images for: CB2-mediated attenuation of nucleus pulposus degeneration via the amelioration of inflammation and oxidative stress in vivo and in vitro
Source: Mol Med. 2021 Aug 19;27:92. doi: 10.1186/s10020-021-00351-x (PMC8377943; doi:10.1186/s10020-021-00351-x)

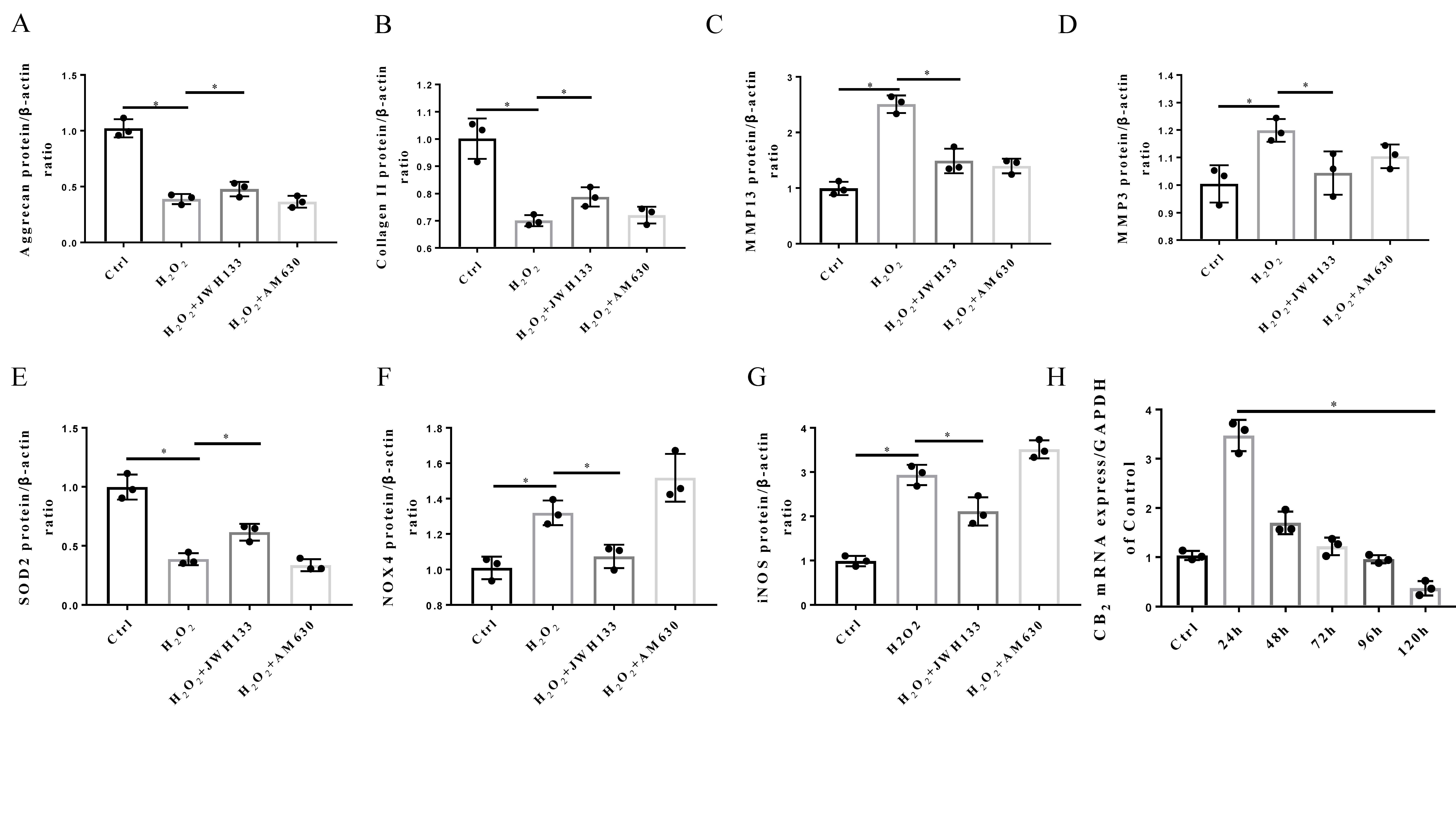

Supplement: Supplementary file 1 — Additional file 1: Figure S1. Quantification of western blot. A-D: quantification of degenerative markers in western blot (Fig. 3, E), E-G: quantification of oxidative stress markers (Fig. 5, F), H: CB2 mRNA expression level in different time points (0, 24, 48, 72, 96, 120 h). (*p < 0.05). [file 10020_2021_351_MOESM1_ESM.tiff]

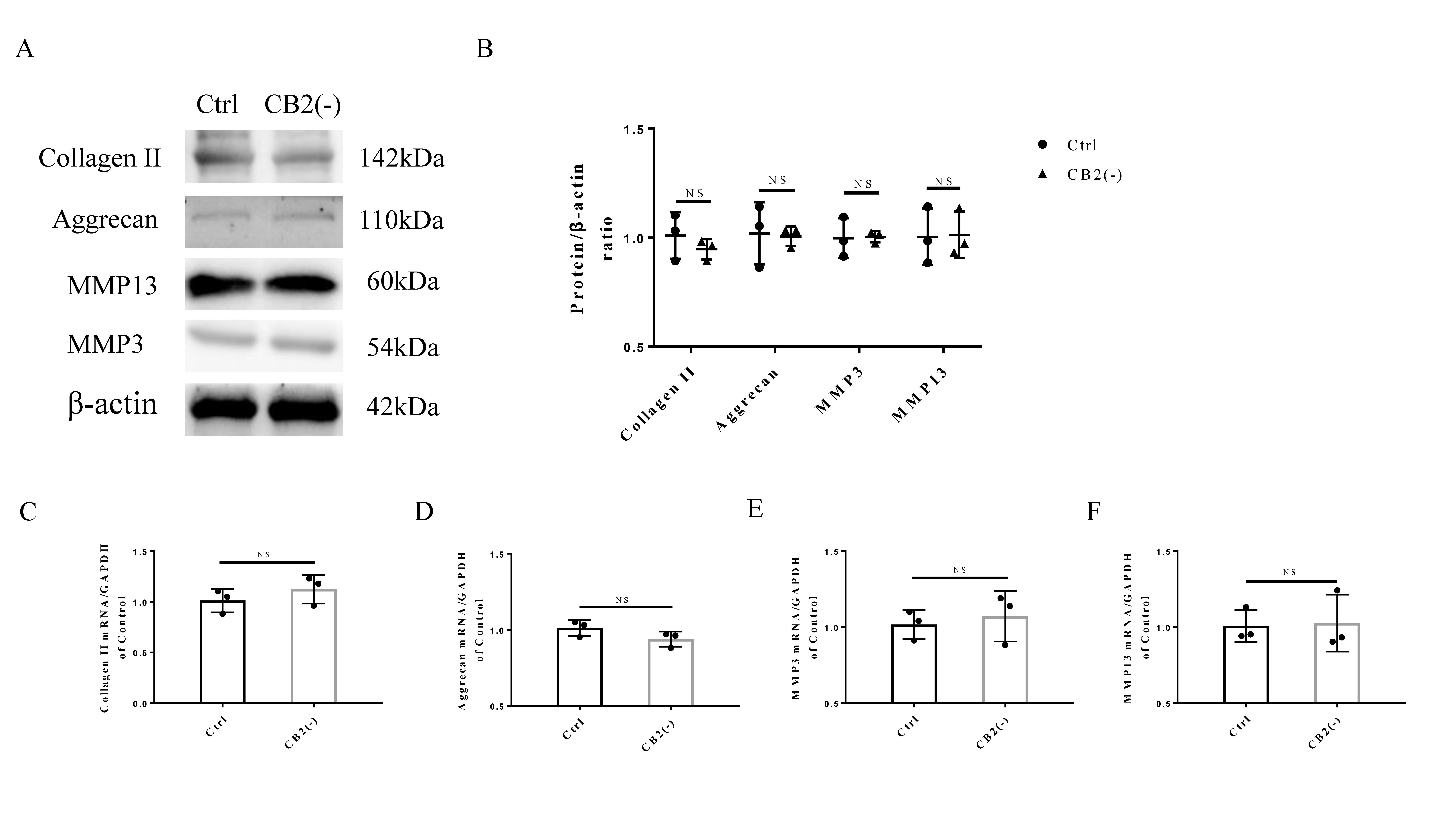

Supplement: Supplementary file 2 — Additional file 2: Figure S2. Inhibition of CB2 do not cause phenotypic change of NPCs. A: western blot results for collagen II, aggrecan, MMP3, MMP13. B: quantification of western blot. C-F: mRNA expression level of collagen II, aggrecan, MMP3 and MMP13. (NS: p > 0.05). [file 10020_2021_351_MOESM2_ESM.tiff]
